# Geochemical Baseline Values Determination and Evaluation of Heavy Metal Contamination in Soils of Lanping Mining Valley (Yunnan Province, China)

**DOI:** 10.3390/ijerph16234686

**Published:** 2019-11-25

**Authors:** Zuran Li, Judith Deblon, Yanqun Zu, Gilles Colinet, Bo Li, Yongmei He

**Affiliations:** 1 College of Horticulture and Landscape, Yunnan Agriculture University, Kunming 650201, China; lizuran@foxmail.com; 2 BIOSE Department, Soil-Water-Plant Exchanges, University of Liège, Gembloux Agro-Bio Tech, 2 Passage des Déportés, 5030 Gembloux, Belgium; judith.deblon@uliege.be; 3 College of Resources and Environment, Yunnan Agricultural University, Kunming 650201, China; libo@ynau.edu.cn (B.L.); heyongmei@ynau.edu.cn (Y.H.)

**Keywords:** heavy metals, contamination, risk evaluation, geochemical baseline values, Lanping mining valley (China)

## Abstract

The largest lead/zinc mine in China is located in Lanping mining valley. The real impact of mining activity on the Lanping mining valley has not been studied to date. This study aims to characterize the geochemical baseline values and risk assessment of heavy metal contamination in soils of a study area located in the Lanping mining valley including upstream, mining and downstream areas. The results showed that the mean soil pH value was 6.8, and organic matter was 34.3%, in surface layer of the mining area. The mean soil pH value in the upstream and downstream areas was less than 5.5. The concentrations of Pb and Zn in the mining area were 56 and 47 times above the world average, the concentrations of Pb, Zn and Cd in the upstream area were six, seven, and six times above the world average, and the concentrations of Pb, Zn and Cd in the downstream area were eight, eight, and 18 times above the world average, respectively. The proposed geochemical baseline values of Pb, Cu, Zn and Cd were 169.93, 31.81, 569.06 and 4.13 mg·kg^−1^, respectively. The pseudo total and ethylene diamine tetraacetic acid (EDTA)-extractable concentrations of Pb, Zn and Cd showed similar tendency as follows: mining area > downstream area > upstream area. The contamination degree with the geoaccumulation index (I_geo_) and the improved Nemerow index (I_IN_) in the upstream and mining areas was non-contamination or slight contamination with low or moderate risk with the individual ecological risk index (E_r_) and the comprehensive potential ecological risk index (RI), although moderate or heavy contamination with pollution factor (Pi) and the Nemerow index (I_N_). The contamination degree with I_geo_ and I_IN_ in the downstream area was non-contamination or extreme contamination with low or extreme risk with E_r_ and RI. The results suggest that the I_IN_ should be recommended to assess the soil contamination of heavy metals and the geochemical baseline values would be important for the environmental management and remediation of soils contaminated by heavy metals.

## 1. Introduction

Soil heavy metal pollution is a common environmental problem of global concern [1,2,3]. The main causes of soil pollution include human activities such as industry, mining, agriculture and high background values of soil. The soil environment is not optimistic in China. Among the 1672 soil sites surveyed in 70 mining areas in China, 33.4% exceeded the standard values of pollutants, including cadmium, lead and arsenic. The soil around the nonferrous metal mining area was seriously polluted by cadmium, arsenic and lead (National Soil Pollution Survey Bulletin, 2014). It has brought serious pollution to the soil ecological environment of the mining area and surrounding farmland [4]. It has been reported that a large amount of As, Cd, Cu, Pb and Zn entered into the lake, sediments and aquatic animals near the abandoned gold mining area in British Columbia, Canada [5]. It was found that the exploitation of the Dashkasan gold deposit in Iran resulted in serious pollution of the soil by As, Cd, Hg and Sb. Heavy metal pollution was found in water, soil and plants near the mining and smelting areas of the Tongguan gold mine in Shaanxi Province, the Dabaoshan mine in Guanzhou Province, the Huludao zinc smelting plant in Liaoning Province, the Huize lead-zinc mine in Yunnan Province, the Lanping lead-zinc mine in Yunnan Province, and copper mine in Nulasai, Xinjiang Autonomous Region, China [6,7,8,9,10,11,12]. Heavy metals in soil are transported into the surrounding ecosystem through atmosphere and precipitation and accumulated in the human body along the food chain, affecting human metabolism and body functions. Soil heavy metals have many realistic and potential risks to the ecological environment and human health.

The determination of environmental geochemical baselines, which should be different for each heavy metal in geologically different regions, is necessary to understand the situation of the environment and to provide soil quality standard values in the evaluation of contaminated soils and environmental risk [13,14]. The value of the geochemical baseline could be calculated with the addition of the arithmetic or geometric mean at twice the value of the standard deviation, considering the diffuse entry of these elements into soils [3,15,16]. Many studies on soil heavy metal contamination have been carried out in the Pb and Zn mine area in China, and the environmental geochemical baseline, which could be an important aid to the location of contaminated areas and to the recognition of anthropogenic inputs, is still lacking.

There are many methods to evaluate the degree of soil heavy metal contamination related to environmental and human health issues. The evaluation methods include index methods, model index methods, human health risk assessment methods, a quantitative analysis of available and total heavy metal concentrations, and a geostatistics-based method. The index method mainly includes the Nemerow index (I_N_), enrichment factor, geoaccumulation index (I_geo_), individual ecological risk index (E_r_^i^) and potential ecological risk index (RI). The model index method mainly includes a fuzzy mathematics model, a grey clustering model, and an analytic hierarchy process [17]. The Nemerow index comprehensively reflects the different effects of various heavy metals on soil, but factors with high concentrations may be artificially exaggerated or factors with low concentrations may be reduced [18]. The influence of geological background is considered with I_geo_, but not comparing the environmental quality between elements or region [19]. E_r_^i^ and RI introduce a toxicity response coefficient to link the environmental ecological effect of heavy metals with toxicology, providing a basis for environmental improvement and scientific reference for human health [20]. The evaluation based on the bio-available fraction and total concentrations of heavy metals reflects the real risk and potential risk of pollutants. The results of the comprehensive evaluation of heavy metal pollution were consistent by using I_geo_ and RI in the Gongchangling iron mine in Liaoning Province, China [21]. The reasonable assessment of risks to the ecological environment and human health with soil heavy metals is of great value and significance [2]. It is necessary to combine different evaluation methods to assess the heavy metal pollution of soil and provide geochemical baseline values.

The hypothesis of this study is that soil heavy metal concentrations around lead and zinc mines, influenced by geochemical baseline values and activities of mining and smelting, have transferred to the surrounding ecosystem and resulted in potential human health risks. This study is of great significance in the ecological restoration of the heavy metal pollution of soils around the lead and zinc mining area.

## 2. Materials and Methods

### 2.1. Study Area

The investigation areas were located in Lanping County, Yunnan Province, China, in the central area of a world natural heritage site—Three Parallel Rivers of Nujiang, Langchanjiang and Jinshajiang—at latitude 26°22′4″ N, longitude 99°23′10″ E and elevation 2459 m, in a low-latitude monsoon climate, with an annual average temperature of 11.7 °C and an annual precipitation of 1002.4 mm.

The Jinding deposit is located to the north of the Lanping mining valley [22]. It represents the largest and youngest land-based lead and zinc deposit in Asia and the second largest in the world [23,24]. The Jinding deposit contains a reserve of 2.64 million tons of lead and 12.84 million tons of zinc. This deposit is 50 to 1450 m in length, 10 to 102 m in width and 215 to 1360 m in depth [25]. The Lanping mining valley is composed of silt-mudstone, sandstone, siltite, arenite and conglomerates. Sandstone, limestone, mudstone, siltstone and gypse at the Tertiary level are the composition of the Lanping mine valley. These rocks are 16 geological formations since the Triassic era [25]. Lead and zinc are hidden in tertiary limestone and cretaceous sandstone-like rocks [25]. The metal ores associated with it are sphalerite, galene, pyrite and marcassite [26]. There are many symbiosis and accompanying minerals such as cadmium, thallium, celestite, silver, sulfur and gypsum. The Jiayashan section of the Jinding mining area is mainly used for strip mining.

### 2.2. Soil Sampling and Treatment

**Figure 1:**
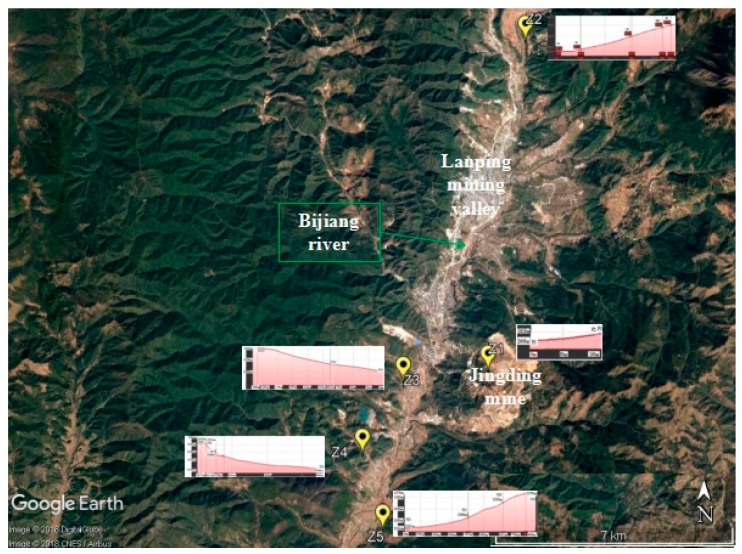
Location of soil sampling.

The study area is 15 km in length and is between 2220 and 2641 m above sea level. A total of 35 samples were collected from five distinct areas. The first area corresponds to the Jinding mine (Z1, mining area). The second (Z2, upstream area) is the slope upstream of the mine, which is 9.57 km from the mine. The third zone (Z3) represents a slope 2.54 km downstream from the mine. The fourth zone (Z4) corresponds to the slope next to the village of Guanping, downstream from the foundry industry, separated by a distance of 4.35 km from the mine. Finally, the fifth area (Z5) sampled corresponds to the slope next to the village “XinJing” which is separated by a distance of 5.50 km from the mine. The downstream area includes Z3, Z4 and Z5 (Figure 1).

For the Z1, Z3, Z4 and Z5 slopes, three profiles (slope summit/middle/bottom) have been dug along the topo-sequence to characterize the distribution of soils, in addition to Z4 and Z5 with another profile in the cultivated land. For the Z2 slopes, two profiles and another three surface layer samples of cultivated soil have been sampled. There were sixteen pairs of soil samples of surface layer–subsurface layer and another three soil samples from the surface layer.

The soil samples were passed entirely through a 2 mm sieve for the determination of pH value and a 0.25 mm sieve for the determination of heavy metal concentrations and organic matter content after air-drying.

### 2.3. Analytical Methods

#### 2.3.1. pH (KCl) Value Determination

pH measurement involved weighing 5 g of soil and adding this to 12.5 mL of distilled water and 12.5 mL of KCl solution (0.67 M). These solutions were then stirred in a rotary mixer at 300 revolutions per minute for 30 min. The pH (KCl) of the supernatant was measured with a pH meter [27].

#### 2.3.2. Organic Matter (OM) Content Determination

The Walkley–Black method was used to quantify the humus content in the soil [27]. This is based on an oxidation reaction of organic carbon by potassium bichromate (K_2_Cr_2_O_7_ (0.8 M)) in an acidic environment (H_2_SO_4_ (95%)). In the presence of a colored indicator, which is a solution of monohydrate o-phenanthroline and ferrous sulphate, potassium bichromate turns from red to green depending on the amount of organic carbon in the soil.

The organic matter content is determined by the following equation:

OM (%) = c (FeSO4)(V0 − V)(1 + 0.01W0) 0.003 × 1.724 × 1.1 × 100/M0

where OM is the organic matter content (%); c(FeSO_4_) is the concentration of ferrous sulphate (mol·L^−1^); V0 is the volume of ferrous sulphate used as a blank (mL); V is the volume of ferrous sulphate used as a soil sample (mL); M0 is the weight of the soil sample (g); W0 is the water content of the soil sample (%).

#### 2.3.3. Pseudo Total Heavy Metal Concentration Determination

Approximately 0.500 g of the soil sample was filtered through a 0.25 mm sieve and accurately weighed before placing into a 150 mL flask. A little water was then added with 10 mL of aqua regia (HNO3: HCl = 1:3) before being left to stand for 24 h. Then, the mixture was heated at a temperature of 140–160 °C, until the brown smoke being let off disappeared. After cooling, 5–10 mL of perchloric acid was added, and the mixture was heated until it turned gray-white in color. After cooling to room temperature, the mixture was filtered using a 50 mL volumetric flask with 0.45 µm filter. The pseudo total heavy metal concentration was determined using an atomic absorption spectrometer (Thermo Scientific ICE 3000, Waltham, MA, USA). The determination procedures were carried out using a blank standard and subsection curve fitting. The analytical limits of Cd, Pb, Zn and Cu detection were 0.005, 0.020, 0.002 and 0.004 ng·mL^−1^, respectively. The operating parameters of the analytical instrumentation included a gas flow rate of 1.2 L·min^−1^, a burner height of 7 mm and an atomizer lift time of 4 s; and the wave length for Cd was 228.8 nm, Pb 283.3 nm, Zn 213.9 nm and Cu 324.8 nm.

#### 2.3.4. Ethylene Diamine Tetraacetic Acid(EDTA)-Extractable Heavy Metal Concentration Determination

This analysis consisted of adding 5 g of soil to 25 mL EDTA (0.05M), buffered to pH 9. The mixture was then placed in a rotating agitator for 2 h at a rate of 300 revolutions per minute. The EDTA-extractable heavy metal concentrations were determined by using the atomic absorption spectrometer (Thermo Scientific ICE 3000, Waltham, MA, USA).

### 2.4. Calculation Methods

Pi = Ci/Si(1)

where Pi is the pollution factor; Ci is the concentration of i metal in a sample; Si is the secondary standard value of i metal based on the Environmental Quality Standard for Soils (GB15618-1995). Pi is classified as class 0 (Pi <1) non-contamination, 1 (1 < Pi < 2) slight contamination, 2 (2 < Pi < 3) low contamination, 3 (3 < Pi < 5) moderate contamination and 4 (Pi > 5) heavy contamination.

I_N_ = √[(Pi_max_^2^ + Pi_ave_^2^)]/2 (2)

where I_N_ is the Nemerow index; Pi_max_ is the maximum Pi value of all metals in a sample, and Pi_ave_ is the mean of the Pi. I_N_ is classified as class 0 (I_N_ < 0.7), 1 (0.7 < I_N_ < 1), 2 (1 < I_N_ < 2), 3 (2 < I_N_ < 3) and 4 (I_N_ > 3), which is the same as for Pi.

I_geo_ = log_2_(C_i_/1.5B_i_) (3)

where I_geo_ is the geoaccumulation index; C_i_ is the measured concentration of the i metal examined in the soil; B_i_ is the background level of the i metal. The factor 1.5 was used to correct possible variations in the background values of a particular metal in the environment. I_geo_ is classified as class 0 (I_geo_ < 0) non-contamination, 1 (0 < I_geo_ < 1) slight contamination, 2 (1 < I_geo_ < 2) low contamination, 3 (2 < I_geo_ < 3) moderate contamination, 4 (3 < I_geo_ < 4) heavy contamination, 5 (4 < I_geo_ < 5) high contamination and 6 (I_geo_ > 5) extreme contamination.

I_IN_ = √(I_geomax_^2^ + I_geoave_^2^) (4)

where I_IN_ is the improved Nemerow index; I_geomax_ is the maximum I_geo_ value of all metals in a sample, and I_geoave_ is the mean of the I_geo_. I_IN_ is classified as class 0 (I_IN_ < 0.5), 1 (0.5 < I_IN_ < 1), 2 (1 < I_IN_ < 2), 3 (2 < I_IN_ < 3), 4 (3 < I_IN_ < 4), 5 (4 < I_IN_ < 5) and 6 (I_IN_ > 5), which is the same as for I_geo_.

E_r_^i^ = T_r_^i^ × C_f_^i^ (5)

where E_r_^i^ is the individual ecological risk index; T_r_^i^ is the toxicity coefficient of each metal—the standard values of which are Pb = 5, Cu = 5, Zn = 1 and Cd = 30 [2,28]; C_f_^i^ is the contamination factor (C_f_^i^ = C_i_/B_i_), where C_i_ is the measured concentration of the pollutant and B_i_ is the level of geological background. E_r_^i^ is classified as class low risk of contamination (E_r_^i^ < 40), moderate risk of contamination (40 < E_r_^i^ < 80), considerable risk of contamination (80 <E_r_^i^ < 160), high risk of contamination (160 < E_r_^i^ < 320) and extreme risk of contamination (E_r_^i^ > 320).

RI = Σ^m^_i = 1_E_r_^i^ (6)

where RI is the comprehensive potential ecological risk index. RI is classified as class low potential ecological risk (RI < 150), moderate potential ecological risk (150 < RI < 300), considerable potential ecological risk (300 < RI < 600) and extreme potential ecological risk (RI > 600).

### 2.5. Data Statistical Analysis

Data were analyzed by using plot units and descriptive statistics in Excel 2010. Box plots and correlation analysis between heavy metal concentrations were determined by using Statistical Product and Service Solutions (SPSS) 19.0. Significance thresholds were set at *P* < 0.05 (significant) and *P* < 0.01 (highly significant). Only significant relationships are reported.

## 3. Results and Discussion

### 3.1. Soil pH Value and Organic Matter Contents

Statistical data on the pH and organic matter contents of the surface layer (0–20 cm) soils and subsurface layer (40–60 cm) soils of the mining area, upstream and downstream are presented in Table 1. The mean pH value in the surface layer of the mining area was higher than the other two sites—it was neutral. The mean pH values in the upstream and downstream areas were less than 5.5—it was mainly acid soil. The pH values in the subsurface layer were less than 6.0—it was acid soil. The low pH value of soil may be related to the acidification of soil caused by dust removal of acidic gas in the smelting process of lead and zinc ore [12,29]. The availability of heavy metals in acidic soils increased the uptake of heavy metals by plants.

The organic matter (OM) contents in surface and subsurface layers of the mining area were higher than those in the downstream area and the upstream area. The higher organic matter content in the mining area may be due to the higher heavy metal concentration in the soil, which leads to a decline in microbial activity and the inability to decompose organic matter rapidly [30,31]. The content of soil organic matter in the upstream and downstream areas was related to the application of organic fertilizer and straw returning to the field during agricultural production.

### 3.2. Concentration of Heavy Metals in Soils

The average values of the pseudo total heavy metals obtained from the statistical analysis of the soils of three sites can be seen in Table 2, where other bibliographical data on levels obtained in other countries have also been attached for purposes of comparison. The kurtosis and skewness were used to understand the distribution characteristics of the data.

In the statistical analysis of this study, the data on the most surface layers (0–20 cm) and subsurface (40–60 cm) layers were treated separately. The surface layers provided more information on the levels of pollution caused by the processes of soil formation and by anthropogenic sources, whereas the subsurface layers exclusively represent the lithogenic contributions, since there is little probability of contamination through atmospheric deposition. It is for this reason that samples of soil in the subsurface horizon were used to determine the level of the natural geological background [2].

The concentrations of Pb, Cu and Cd were above the world average. The Zn concentration was equivalent to the world average. The highlight of the mining area was the high concentration of Cd in soils, since it had a value higher than the world’s top-ranking sample (Table 2).

As for the values obtained from soils in the mining area, it is worth noting the abnormally high values across all samples for Pb concentration, which compared to the world average of 15 mg·kg^−1^ and the global range between 2 and 200 mg·kg^−1^ [32], although the range has reached as high as 1500 mg·kg^−1^ [33]. Cu concentration did not exceed the values of the world average (12 mg·kg^−1^). Zn concentrations obtained in this study exceeded the world average values of 40 mg·kg^−1^. In some soil samples, values higher than 2600 mg·kg^−1^ were reached. Of the soil samples collected in the mining area, Cd concentrations exceeded the world average (0.4 mg·kg^−1^), with concentrations (26.33 mg·kg^−1^) up to 66 times higher than most other countries in the world, and the range reached as high 37.24 mg·kg^−1^. It was noted that the concentrations of Pb and Zn were 56 and 47 times above the world average. To summarize, from the study of heavy metals in soils in the mining area, the geochemical anomalies in Pb, Zn and Cd concentrations were highlighted.

In relation to the values obtained in the soils of the upstream area, Pb, Cu, Zn and Cd concentrations of soil samples exceeded the world average values. However, the concentrations of Cu were slightly higher than those obtained in other countries. The concentrations of Pb, Zn and Cd obtained from the upstream area were six, seven, and six times higher, respectively, than the levels obtained in soils from other parts of the world.

Pb, Cu, Zn and Cd concentrations of soil samples from the downstream area exceeded the world average values. The concentrations of Cu were slightly higher than those obtained in other countries. The concentrations of Pb, Zn and Cd obtained from the upstream area were eight, eight, and 18 times higher, respectively, than the levels obtained in soils from other parts of the world.

Because some risk assessments of agricultural soil were focused on Pb, Cu, Zn and Cd contamination in Lanping recently, four heavy metals only were measured [34,35,36]. However, other heavy metals such as Hg, Co, Ni, As and Se may also show high concentrations that need to be addressed in the future.

#### 3.2.1. Box Plots of Heavy Metal Concentrations in Soils

**Figure 2:**
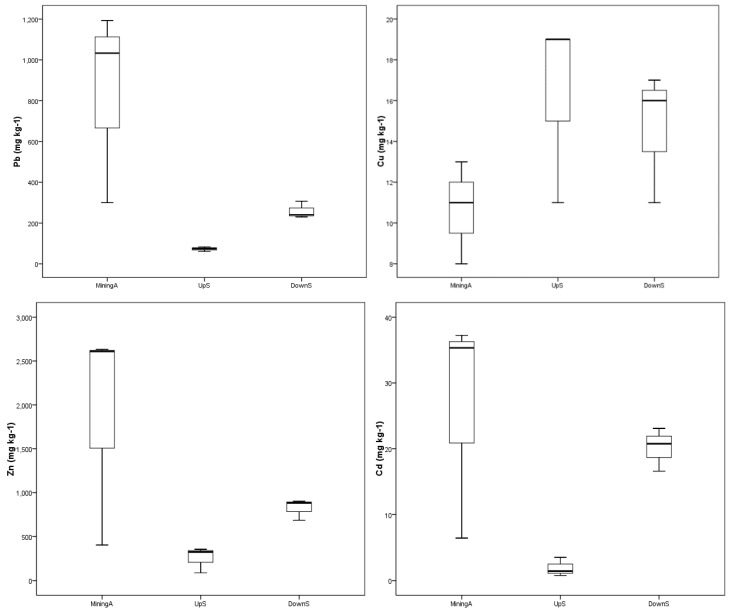
Box plots of heavy metal concentrations.

The box plots in Figure 2 show a summary of the basic statistics of the concentrations of heavy metals studied in the soils of the Lanping mining valley. When comparing the concentrations of heavy metals in the three areas studied, it can be described better graphically through several box plots. In the upstream area, Pb, Zn and Cd concentrations were lower with respect to the mining and downstream areas, which was justified by the null or weak contamination of Pb and Zn in soils of the upstream area, given the lack of mining activities when compared to areas with extensive and mining activities such as mining area and water pollution along Bijiang river [34,35]. Some investigation showed that the concentrations of Pb, Zn and Cd in the cultivated soil along the Bijiang river were the highest surrounding mining areas, and then decreased, presenting a zonal distribution pattern [36]. Only the upstream area had no significant different Cu concentrations compared with the mining and downstream areas, as three sites belong to the same Pb and Zn metallogenic belts.

#### 3.2.2. Geochemical Baseline Study of the Soils of the Upstream Area 

To calculate the geochemical baseline, the geometric mean was used with a normal logarithmic heavy metal distribution—in cases where the variables were strongly asymmetric and their transformation could not be adjusted to normal, then the median was used [37]. The normal distribution of metal concentrations was investigated by performing the Kolmogorov–Sminov test, although this can also be inferred by the existence of high kurtosis values.

In the case of soils of the upstream area, concentrations of heavy metals from all the horizons were used to calculate the baseline, which was conducted by adding the geometric mean to twice the standard deviation [3,38,39] (Table 3). The proposed baseline values of Pb, Cu, Zn and Cd were 169.93, 31.81, 569.06 and 4.13 mg·kg^−1^, respectively.

There were few studies about the soils in the upstream area located at the north part of the mining area. Ref. [34] sampled soils at two points (Guodeng village and Taoshu village) in the upstream area as reference points, which was consistent with the upstream site in our study. Therefore, it is feasible and reasonable to select heavy metal concentrations of soils in the upstream area for geochemical baseline estimation. The geochemical baseline values for Pb, Zn and Cd were higher than natural geological background values in the upstream and downstream areas and lower than those in the mining area. The geochemical baseline values could be used to estimate the background values for trace metals in soils, related to pre-industrial activities and normalized using soil properties, such as pH, texture, or organic matter [40,41]. Comparing with the risk screening values (Pb 100, Cu 150, Zn 200 and Cd 0.4 mg·kg^−1^) and risk intervention values (Pb 500 and Cd 2.0) for soil contamination of agricultural land (GB15618-2018), the proposed geochemical baseline values of Pb, Zn and Cd were higher than the risk screening values, and even higher than the risk intervention value for Cd. This suggested that the regional geochemical baseline values would be more important for soil quality and risk control. The risk screening values and the risk intervention values should be adjusted according to soil characteristics and regional condition. Considering the high Cd geochemical baseline value, it is necessary to take more samples and improvement. Because the soils of the upstream area may have been affected by the input of acidic dust deposition as evidenced by the low soil pH, the baseline values of heavy metals in soils of the upstream area should be studied more further.

The application of the regional geochemical baseline values proposed in this study will allow the rapid identification of sites that could be affected by pollution processes due to current mining exploitation in the area [3]. In addition, this information will also be very useful for a restoration plan to try to reproduce the environmental conditions that existed prior to mining [39,42].

#### 3.2.3. EDTA-Extractable Concentrations of Heavy Metals

Of the soil samples collected in the mining area, EDTA-extractable Pb and Cu concentrations accounted for 63–84% and 63–100% of the pseudo total concentrations, respectively. EDTA-extractable Zn and Cd accounted for 4–28% and 15–35% of the pseudo total concentrations. This indicated that the Pb and Cu had high mobility, while Zn and Cd had low mobility in the mining area (Table 4).

Based on the values obtained in the soils of the upstream area, EDTA-extractable Pb, Cu, Zn and Cd concentrations of soil samples were 33–57%, 16–60%, 0–21% and 0–38% of the pseudo total concentrations, respectively. EDTA-extractable Pb, Cu, Zn and Cd concentrations of soil samples from the downstream area were 36–100%, 33–87%, 2–80% and 25–56% of the pseudo total concentrations, respectively. EDTA-extractable heavy metal concentrations were as follows: mining area > downstream area > upstream area, except for Cu. The mobility of Pb and Cu in surface soils was higher than Zn and Cd.

The available Pb and Cd concentrations (water soluble, ion exchangeable, carbonate bound, and humic acid bound) were high in cultivated soils along the Bijiang river watershed. Plants absorb Pb and Cd into the food chain, resulting in threats to human health, especially in acid soil with a low pH value [7,8,34]. Zn was mainly bound to Fe and Mn oxides or a residual fraction with weak migration ability, which was basically consistent with the results of this study [34]. Pyrite is rich in the Lanping Pb and Zn mine and coexists with galena. The weathering speed of galena is accelerated due to acid production of pyrite weathering [8,43]. The increase in contents of the active fractions of heavy metals poses potential ecological risks in the Lanping Pb and Zn mining areas and the Bijiang river watershed.

#### 3.2.4. Correlation Analysis

Positive correlations between Pb, Cu, Zn and Cd concentrations in surface (*p* < 0.01, *N* = 19) and subsurface layers (*p* < 0.01, *N* = 16) were observed (Table 5). There were positive relationships between pseudo total and EDTA-extractable concentrations of Pb, Cu, Zn and Cd in the surface horizon. The concentrations of heavy metals in the surface soil were consistent with that in the subsoil to some extent. Positive correlations between pH value and concentrations of Pb, Zn and Cd in surface (*p* < 0.01, *p* = 19) and subsurface layers (*p* < 0.01, *N* = 16) were observed. As the surface layer of heavy metals accumulated, the EDTA-extractable Pb, Cu, Zn and Cd in the surface soil would leach and migrate to impact on the subsoil and groundwater. With the decrease in soil pH value, concentrations of available Pb, Zn and Cd gradually increase, and the migration capacity of heavy metals increases, which increased the ecological risk to the surrounding water and environment [9]. The oxidation and residual fractions of heavy metal increased with an increase in pH value [44]. Therefore, soil pH value in this region has a great influence on soil heavy metal concentrations.

### 3.3. Assessment of Environmental Risks: Pollution Rates

The states of heavy metal contamination in soils from the areas studied in this work were evaluated using different quantitative contamination rates.

#### 3.3.1. The Pollution Factor and Nemerow Index

Using the pollution factor and Nemerow Index to determine pollution levels in the mining area showed that all soil samples were contaminated by Pb, Zn, and Cd. The pollution factor of Cu was less than 1 (Table 6). These data showed that soils in the mining area were highly contaminated. Similar reports showed that the concentrations of Pb, Cd and Zn in the soil of the Jinding mining area exceeded 67%, 97% and 66% of the third standards values of soil environmental quality (GB15618-1995, Zn 500, Cd 1, Pb 500 mg kg^−1^) and showed high contamination [7,8].

In the upstream area, the pollution factor showed that the soils were contaminated by Zn (60%) and Cd (100%). The I_N_ showed that the soils were moderately contaminated 20% and heavily contaminated 80%. In the downstream area, the pollution factor showed that the soils were contaminated by Pb (9%), Zn (45%) and Cd (100%). The I_N_ showed that the soils had low contamination 9%, moderate contamination 9% and heavy contamination 82%, and concentrations of Pb, Cd and Zn in the cultivated soils around the mining area exceeded 67–80%, 49–92% and 94–100% of the secondary standards values of soil environmental quality (GB15618-1995) [34,35,36]. Huang [36] reported that the average value of the Nemerow Index I_N_ of cultivated soil along Bijiang river was 17.69—in which, 5.71% of soils was non-contaminated or slightly contaminated, and 94.29% of soils was moderately contaminated (2.85%) and heavily contaminated (91.17%). The cultivated soils in the downstream area were mainly heavily contaminated.

#### 3.3.2. The Geoaccumulation Index and the Improved Nemerow Index

The I_geo_ for the four heavy metals studied are summarized in Table 7. The I_geo_ of the analyzed heavy metals allowed the analysis of the single-factor contamination index to evaluate the presence of each individual metal and its level of contamination in the study area.

In the mining area, considering the I_geo_, most soils were included in Class 0 (I_geo_ ≤ 0) and Class 1 (0 < I_geo_ ≤ 1)—that is, non-contaminated to slightly contaminated, with 67.7% and 25.0% of the samples, respectively, and only some soils in Class 3 (2 < I_geo_ ≤ 3), which were moderately contaminated by Pb in 8.3% of the samples. In the upstream area, samples of the soils studied were included in Class 0 and Class 1, with non-contaminated to slightly contaminated consisting of 75% and 25% of the samples, respectively. In the downstream area, the soils, were classified as Class 0 (22.0%), Class 1 (29.3%, Class 2 (12.2%), Class 3 (9.8%), Class 4 (19.5%), Class 5 (2.4%) and Class 6 (4.9%), which ranged from non-contaminated to extremely contaminated.

By considering the I_geo_, the levels of contamination (from low to high) of Zn and Cd in the surface horizons of the soils was as follows: downstream > upstream > mining area. The level of Cu in three sites and level of Pb in the mining and upstream areas was non-contaminated and level of Pb in the downstream area was slightly contaminated.

In the mining area, when considering the I_IN_, the soils ranged from non-contaminated to moderately contaminated: 33.3% of samples were in Class 0 (I_IN_ < 0.5), 33.3% in Class 1 (0.5 ≤ I_IN_ < 1), and 33.4% of soils in Class 3 (2 ≤ I_IN_ < 3). However, it should be pointed out that the heavy degree of contamination indicated by this index may be due to background values. In the upstream area, all soil samples were classified as slightly contaminated Class 1 based on the I_IN_. The soils of the downstream were considered as slightly to highly contaminated: Class 1 (9.1% of the samples), Class 2 (27.3%), Class 3 (27.3%), Class 4 (9.1%) and Class 5 (27.3%). The degree of contamination in the downstream area may be due to anthropogenic processes (atmospheric deposition of metals, medium or long distance), which led to an increase in geological concentrations in the soils of the downstream area. The main external sources of heavy metals in the soils of this area are through diffuse pollution, and wet and dry deposition caused by metallic mining and the processing of minerals from the mining area that has led to the accumulation of heavy metals due to long-term use. By using the I_IN_, heavy metal contamination levels (from low to high) from soil surface horizons are as follows: downstream area > mining area > upstream area. The degree of assessment in the downstream area was consistent with I_IN_ and I_N_. However, there was a great difference in calculating the percentage and the degree of contamination in soils based on different contamination indices, especially in the mining and upstream areas. I_IN_ was much more accurate in assessing the environmental risks of heavy metal contamination compared with I_N._ I_IN_ excludes the influence of a high geological background value on the evaluation and true reflection of the extent and source of soil contamination, making the conclusion more credible and objective.

#### 3.3.3. Potential Ecological Risk Index

The individual ecological risk index (E_r_^i^) evaluates the toxicity of trace elements in sediments and has been extensively applied to soils. Soils contaminated by heavy metals can cause serious ecological risks and negatively impact human health due to various forms of interaction (agriculture, livestock, etc.), through which highly toxic heavy metals can enter the food chain. The excessive accumulation of heavy metals in agricultural soils can affect the quality and safety of food and further increase the risk of serious diseases (cancer, kidney, liver damage, etc.), as well as impact ecosystems, thus combining environmental chemistry with biological toxicology and ecology.

The comprehensive potential ecological risk index (RI) reflects the general situation of pollution caused by the simultaneous presence of the four heavy metals (Table 8).

Considering the individual ecological risk index (Er), the levels of the heavy metal contamination of the surface horizons of soils of the mining areas were considered to be of low contamination risk (Er < 40), with only three soil samples (25.0%) with a risk of moderate contamination (40 ≤ Er < 80) from Cd and Pb.

In the upstream area, the majority of the samples (87.5%) were considered to have a low risk of contamination (Er < 40); one sample (12.5%) was considered to have a moderate contamination risk (40 ≤ Er < 80) from Cd.

In the downstream area, it was also shown that the majority of the samples (66.7%) were considered to be at low risk of contamination (Er < 40); a small percentage (14.3%) of samples were considered to have a moderate contamination risk (40 ≤ Er < 80) from Pb and Cd; 4.8% of samples presented a considerable risk of contamination (80 ≤ Er < 160) form Pb and Cd; and 14.2% of soil samples had an extreme risk of contamination (Er > 320) from Cd.

If we consider the individual ecological risk index (Er), the levels of contamination (from lowest to highest) by heavy metals from the surface horizons of soils were as follows: downstream area > mining area > upstream area. Some studies showed that the Er of Cu and Zn of cultivated soils in the downstream area along Bijiang river was less than 40, i.e., at low risk of contamination; the Er of Pb was more than 40 in some samples, which was a moderate contamination risk. Cd contributed to the extreme risk of contamination. The contribution of various heavy metals to Er was Cd > Pb > Zn > Cu in sequence [36].

To calculate the potential response rate to the toxicity of all the studied heavy metals (RI), the contamination levels were as follows: in the mining area and upstream, all soil samples (100%), present low potential ecological risk (RI < 150). In the downstream area, 45.5% of the samples had a low potential ecological risk (RI < 150); 18.2% of samples faced a significant potential considerable ecological risk (300 ≤ RI < 600). In total, 36.4% of samples had an extreme ecological risk of potential contamination (IR > 600).

Furthermore, when considering the potential ecological risk index (RI), the levels of contamination (from low to high) from heavy metals from the surface horizons of soils were as follows: downstream area > mining area > upstream area. Huang [36] reported that the average RI of soils along Bijiang river was 773.38, indicating extreme ecological risk of potential contamination.

Based on the different contamination degrees, the remediation of soils in the Lanping mining valley was recommended for passivation agent application in slightly contaminated soil, intercropping of hyperaccumulator and low accumulation crop variety in moderately contaminated soil, and energy-alternative plants in heavily or extremely contaminated soil.

## 4. Conclusions

Soils were characterized by acid and high organic matter contents, with high concentrations of Pb, Zn and Cd in the Lanping mining valley (China). The average concentrations of pseudo total Pb, Zn and Cd were much higher than the values obtained from other parts of the world. The pseudo total and high EDTA-extractable Pb, Zn and Cd concentrations were as follows: mining area > downstream area > upstream area. The geochemical baseline values of Pb, Zn, Cu and Cd based on soils of the upstream area were 169.93, 569.06, 31.81 and 4.13 mg·kg^−1^, respectively.

Several pollution indices (Pi, I_N_, Igeo, I_IN_, E_r_ and RI) have been used to evaluate soil environmental risk. Data revealed that the soils were non-contaminated or slightly contaminated in the upstream area; non-contaminated or heavily contaminated in the mining area; and slightly contaminated or extremely contaminated in the downstream area, especially with Cd.

Using the Nemerow index I_N_ for environmental risk assessment, the soils of the upstream area were moderately contaminated and heavily contaminated soils; however, using the improved Nemerow index I_IN_, most soils were non-contaminated to slightly contaminated. The results using I_IN_ were consistent with risk assessment using E_r_^i^ and RI. Thus, I_IN_ would be more suitable for assessing the environmental risks of heavy metal contamination.

The results on the environmental database of soils in Lanping mining valley could facilitate the development of management strategies and the remediation of soils contaminated by heavy metals.

## Figures and Tables

**Table 1:** Statistical data on pH and organic matter (OM) contents.

Area	Data	Surface Layer		Subsurface Layer	
		pH (KCI)	OM (%)	pH (KCI)	OM (%)
Mining area	Mean	6.8 ± 0.8	34.3 ± 29.1	5.9 ± 1.6	9.7 ± 8.1
	Range	5.9–7.4	7–65	4.2–7.3	4–19
Upstream	Mean	5.4 ± 1.5	4.9 ± 2.9	4.3 ± 0.8	2.9 ± 2.6
	Range	3.8–7.2	1.9–9.6	3.7–4.9	1–4.7
Downstream	Mean	4.4 ± 0.7	22.1 ± 13.2	4.5 ± 0.9	9.5 ± 13.7
	Range	3.7–5.6	1.7–43.7	3.8–6.6	1.7–49.5

**Table 2:** Statistical analysis of pseudo total heavy metals in the surface horizons of soils/mg·kg^−1^.

**Mining Area**	**Pb**	**Cu**	**Zn**	**Cd**
Mean	842.00	10.67	1881.67	26.33
Geometric mean	717.72	10.46	1404.14	20.38
Median (Me)	1033.00	11.00	2610.00	35.33
Standard deviation (SD)	476.15	2.52	1280.61	17.26
Minimum	300.00	8.00	403.00	6.43
Maximum	1193.00	13.00	2632.00	37.24
Variable coefficient (%)	56.55	23.59	68.06	65.56
Kurtosis	−2.90	−2.79	−3.26	−3.22
Skewness	−0.16	−1.16	−0.07	−0.06
25th percentile	666.50	9.50	1506.50	20.88
75th percentile	1113.00	12.00	2621.00	36.29
Natural geological background (mean in the subsurface horizon)	738.67	11.00	1628.00	18.24
**Upstream**	**Pb**	**Cu**	**Zn**	**Cd**
Mean	90.60	18.80	277.00	2.30
Geometric mean	80.03	18.18	218.45	1.96
Median (Me)	75.00	19.00	323.00	1.90
Standard deviation (SD)	56.24	5.02	186.59	1.34
Minimum	46.00	11.00	88.00	0.78
Maximum	188.00	25.00	526.00	3.88
Variable coefficient (%)	62.08	26.70	67.36	58.11
Kurtosis	3.84	2.20	−1.52	−2.43
Skewness	1.89	−0.77	0.20	0.25
25th percentile	61.00	19.00	94.00	1.44
75th percentile	83.00	20.00	354.00	3.50
Natural geological background (mean in the subsurface horizon)	55.50	24.50	319.00	0.96
**Downstream**	**Pb**	**Cu**	**Zn**	**Cd**
Mean	123.00	14.00	320.09	7.21
Geometric mean	95.12	13.60	203.01	3.53
Median (Me)	67.00	14.00	122.00	3.26
Standard deviation (SD)	94.87	3.44	331.85	8.55
Minimum	47.00	8.00	80.00	0.41
Maximum	307.00	20.00	902.00	23.09
Variable coefficient (%)	77.13	24.54	103.67	118.63
Kurtosis	−0.49	−0.28	−0.42	−0.32
Skewness	1.00	0.00	1.19	1.21
25th percentile	50.00	11.50	91.50	1.44
75th percentile	191.00	16.50	458.00	10.98
Natural geological background (mean in the subsurface horizon)	13.82	13.00	39.18	0.37
World average	15	12	40	0.40
World ranks (Giaccio et al., 2012)	2–200	0.1–200	1–800	0.01–2
World ranks (Monton-Bermea et al., 2009)	1–1500	0.1–250	1–1500	0.01–2

**Table 3:** Statistical analysis of heavy metal concentrations in all horizons of soils of the upstream area and the geochemical baseline (mg kg^−1^).

Upstream Area	Pb	Cu	Zn	Cd
Mean	76.43	21.14	301.86	2.01
Geometric mean	66.11	20.35	252.76	1.74
Median (Me)	61.00	20.00	354.00	1.44
Standard deviation (SD)	51.91	5.73	158.15	1.20
Minimum	41.00	11.00	88.00	0.78
Maximum	188.00	27.00	526.00	3.88
Variable coefficient (%)	67.92	27.09	52.39	59.53
25th percentile	43.50	19.00	208.50	1.29
75th percentile	79.00	26.00	364.00	2.70
Proposed geochemical baseline values	169.93	31.81	569.06	4.13

**Table 4:** Statistical analysis of ethylene diamine tetraacetic acid (EDTA)-extractable heavy metal concentrations in the surface horizons of soils/mg·kg^−1.^

**Mining Area**	**Pb**	**Cu**	**Zn**	**Cd**
Mean	625.33	9.00	143.33	5.67
Geometric mean	527.32	8.43	136.04	4.85
Median (Me)	653.00	10.00	111.00	5.37
Standard deviation (SD)	387.24	3.61	59.50	3.57
Minimum	225.00	5.00	107.00	2.26
Maximum	998.00	12.00	212.00	9.38
Variable coefficient (%)	61.93	40.06	41.51	62.95
Kurtosis	−1.35	−2.60	−0.78	0.27
Skewness	0.28	−0.39	−0.09	0.80
25th percentile	439.00	7.50	109.00	3.82
75th percentile	825.50	11.00	161.50	7.38
**Upstream**	**Pb**	**Cu**	**Zn**	**Cd**
Mean	36.20	7.80	19.80	0.57
Geometric mean	33.52	6.95	−	−
Median (Me)	35.00	9.00	10.00	0.27
Standard deviation (SD)	16.30	3.70	30.12	0.58
Minimum	20.00	3.00	0.00	0.00
Maximum	62.00	12.00	73.00	1.33
Variable coefficient (%)	45.03	47.45	152.12	101.66
Kurtosis	1.34	−1.81	4.51	−2.31
Skewness	1.11	−0.38	2.10	0.61
25th percentile	25.00	5.00	4.00	0.21
75th percentile	39.00	10.00	12.00	1.04
**Downstream**	**Pb**	**Cu**	**Zn**	**Cd**
Mean	87.45	6.91	87.27	2.09
Geometric mean	59.45	6.27	47.48	1.17
Median (Me)	51.00	5.00	54.00	1.18
Standard deviation (SD)	86.75	3.48	90.36	2.16
Minimum	19.00	4.00	13.00	0.23
Maximum	287.00	14.00	245.00	5.75
Variable coefficient (%)	99.19	50.33	103.54	103.33
Kurtosis	1.85	0.22	−0.33	−0.69
Skewness	1.60	1.26	1.02	1.00
25th percentile	30.50	5.00	14.00	0.41
75th percentile	110.50	8.50	124.00	3.37

**Table 5:** Correlation analysis between heavy metal concentrations and soil properties.

*X*	*Y*	Equation	*F*	*R*	*N*	*p*
pH in surface soil	Pb concentrations in surface soil	Y = −648.7 + 174.4X	14.561	0.679	19	0.001
	Zn concentrations in surface soil	Y = −1541.8 + 417.2X	14.799	0.682	19	0.001
	Cd concentrations in surface soil	Y = −19.2 + 5.6X	9.678	0.602	19	0.006
pH in subsurface soil	Pb concentrations in subsurface soil	Y = −975.9 + 238.4X	12.937	0.693	16	0.003
	Zn concentrations in subsurface soil	Y = −1814.6 + 461.0X	11.651	0.674	16	0.004
	Cd concentrations in subsurface soil	Y = −21.2 + 5.3X	12.557	0.685	16	0.003
Pb concentrations in surface soil	Pb concentrations in subsurface soil	Y = 122.5 + 0.8X	95.154	0.934	16	0.000
Cu concentrations in surface soil	Cu concentrations in subsurface soil	Y = 7.5 + 0.5X	11.782	0.676	16	0.004
Zn concentrations in surface soil	Zn concentrations in subsurface soil	Y = 240.7 + 1.0X	105.685	0.940	16	0.000
Cd concentrations in surface soil	Cd concentrations in subsurface soil	Y = 5.9 + 1.1X	22.766	0.787	16	0.000
EDTA-extractable Pb concentrations in surface soil	Pb concentrations in surface soil	Y = −14.5 + 0.76X	312.050	0.974	19	0.000
EDTA-extractable Cu concentrations in surface soil	Cu concentrations in surface soil	Y = 2.1 + 0.37X	5.164	0.483	19	0.036
EDTA-extractable Zn concentrations in surface soil	Zn concentrations in surface soil	Y = 49.2 + 0.05X	5.428	0.492	19	0.032
EDTA-extractable Cd concentrations in surface soil	Cd concentrations in surface soil	Y = 0.4 + 0.21X	173.951	0.954	10	0.000

**Table 6:** The pollution factor (Pi) of heavy metals and the Nemerow Index (I_N_).

**Mining Area**	**Pi**				**I_N_**
	**Pb**	**Cu**	**Zn**	**Cd**	
Mean	2.87	0.13	7.66	47.46	35.10
Median (Me)	3.44	0.13	10.44	58.88	43.61
Standard deviation (SD)	1.47	0.03	4.89	22.60	16.77
Minimum (Min)	1.20	0.11	2.02	21.43	15.78
Maximum (Max)	3.98	0.16	10.53	62.07	45.91
**Upstream**	**Pi**				**I_N_**
	**Pb**	**Cu**	**Zn**	**Cd**	
Mean	0.34	0.33	1.28	6.37	4.75
Median (Me)	0.30	0.38	1.62	6.33	4.66
Standard deviation (SD)	0.17	0.08	0.77	3.34	2.44
Minimum (Min)	0.18	0.22	0.44	2.60	2.03
Maximum (Max)	0.63	0.40	2.10	11.67	8.62
**Downstream**	**Pi**				**I_N_**
	**Pb**	**Cu**	**Zn**	**Cd**	
Mean	0.48	0.26	1.54	20.52	15.06
Median (Me)	0.27	0.26	0.61	10.87	7.97
Standard deviation (SD)	0.36	0.07	1.60	23.22	17.00
Minimum (Min)	0.19	0.16	0.40	1.37	1.05
Maximum (Max)	1.23	0.40	4.51	69.20	50.68

Note: GB15618-1995(Class II): pH < 6.5, Pb 250 mg kg^−1^, Cd 0.3 mg kg^−1^, Cu 50 mg kg^−1^, and Zn 200 mg kg^−1^; pH 6.5–7.5, Pb 300 mg kg^−1^, Cd 0.6 mg kg^−1^, Cu 100 mg kg^−1^, and Zn 250 mg kg^−1^.

**Table 7:** The geoaccumulation index (I_geo_) of heavy metals and the improved Nemerow index (I_IN_).

**Mining Area**	**I_geo_**				**I_IN_**
	**Pb**	**Cu**	**Zn**	**Cd**	
Mean	0.62	−0.56	−0.17	0.01	1.34
Median (Me)	−0.10	−0.69	−0.36	0.11	0.70
Standard deviation (SD)	1.95	0.35	0.44	0.33	1.40
Minimum (Min)	−0.87	−0.83	−0.48	−0.36	0.38
Maximum (Max)	2.84	−0.17	0.33	0.28	2.94
**Upstream**	**I_geo_**				**I_IN_**
	**Pb**	**Cu**	**Zn**	**Cd**	
Mean	−0.03	−2.08	−0.49	0.30	0.91
Median (Me)	−0.03	−2.08	−0.49	0.30	0.91
Standard deviation (SD)	0.65	0.55	0.20	0.78	0.02
Minimum (Min)	−0.49	−2.47	−0.63	−0.25	0.89
Maximum (Max)	0.43	−1.69	−0.35	0.86	0.92
**Downstream**	**I_geo_**				**I_IN_**
	**Pb**	**Cu**	**Zn**	**Cd**	
Mean	2.05	−0.45	1.88	2.73	3.30
Median (Me)	2.42	−0.50	1.34	3.04	3.31
Standard deviation (SD)	1.21	0.44	1.33	1.99	1.91
Minimum (Min)	0.45	−1.03	0.12	0.07	0.96
Maximum (Max)	3.59	0.42	3.84	5.15	5.88

**Table 8:** The individual ecological risk index (E_r_^i^) and comprehensive potential ecological risk index (RI) of the soils studied.

**Mining Area**	**E_r_^i^**				**RI**
	**Pb**	**Cu**	**Zn**	**Cd**	
Mean	21.56	5.18	1.54	46.11	74.39
Median (Me)	6.99	4.64	1.62	48.71	67.70
Standard deviation (SD)	27.76	1.30	0.34	10.10	33.35
Minimum (Min)	4.12	4.23	1.17	34.96	44.88
Maximum (Max)	53.57	6.67	1.82	54.66	110.57
**Upstream**	**E_r_^i^**				**RI**
	**Pb**	**Cu**	**Zn**	**Cd**	
Mean	7.74	0.78	1.08	1.99	72.30
Median (Me)	7.74	0.78	1.08	1.99	72.30
Standard deviation (SD)	3.37	0.11	0.15	1.03	33.53
Minimum (Min)	5.36	0.70	0.97	1.26	48.60
Maximum (Max)	10.12	0.86	1.18	2.71	96.01
**Downstream**	**E_r_^i^**				**RI**
	**Pb**	**Cu**	**Zn**	**Cd**	
Mean	41.34	5.76	8.12	599.67	592.86
Median (Me)	40.00	5.31	3.81	372.50	395.85
Standard deviation (SD)	30.05	2.01	7.51	615.80	645.81
Minimum (Min)	10.22	3.67	1.63	47.31	21.00
Maximum (Max)	90.29	10.00	21.54	1596.92	1698.79
